# Cutting corners: The impact of storage and DNA extraction on quality and quantity of DNA in honeybee (*Apis mellifera*) spermatheca

**DOI:** 10.3389/fphys.2023.1139269

**Published:** 2023-03-03

**Authors:** Ajda Moškrič, Anja Pavlin, Katarina Mole, Andraž Marinč, Jernej Bubnič, Andreja Opara, Marin Kovačić, Zlatko Puškadija, Aleksandar Uzunov, Sreten Andonov, Bjørn Dahle, Janez Prešern

**Affiliations:** ^1^ Department of Animal Production, Agricultural Institute of Slovenia, Ljubljana, Slovenia; ^2^ Department of Biology, Biotechnical faculty, University of Ljubljana, Ljubljana, Slovenia; ^3^ Faculty of Agrobiotechnical Sciences Osijek, University of J.J. Strossmayer, Osijek, Croatia; ^4^ Centre for Applied Life Sciences Healthy Food Chain Ltd., Osijek, Croatia; ^5^ Faculty of Agricultural Sciences and Food, Ss. Cyril and Methodius University in Skopje, Skopje, Macedonia; ^6^ Company for Applied Research and Permanent Education in Agriculture, Skopje, Macedonia; ^7^ Department of Animal Genetics, Swedish University of Agricultural Sciences, Uppsala, Sweden; ^8^ Norwegian Beekeepers Association, Kløfta, Norway

**Keywords:** spermatheca, honeybee, *Apis mellifera*, breeding, preservation method, microsatellite, DNA extraction, patriline

## Abstract

The purpose of our study was to investigate methods of short-term storage that allow preservation, transport and retrieval of genetic information contained in honeybee queen’s spermatheca. Genotyping of the honeybee colony requires well ahead planned sample collection, depending on the type of data to be acquired. Sampling and genotyping of spermatheca’s content instead of individual offspring is timesaving, allowing answers to the questions related to patriline composition immediately after mating. Such procedure is also cheaper and less error prone. For preservation either Allprotect Tissue Reagent (Qiagen) or absolute ethanol were used. Conditions during transportation were simulated by keeping samples 6–8 days at room temperature. Six different storing conditions of spermathecas were tested, complemented with two DNA extraction methods. We have analysed the concentration of DNA, RNA, and proteins in DNA extracts. We also analysed how strongly the DNA is subjected to fragmentation (through amplification of genetic markers ANT2 and tRNA^leu^-COX2) and whether the quality of the extracted DNA is suitable for microsatellite (MS) analysis. Then, we tested the usage of spermatheca as a source of patriline composition in an experiment with three instrumentally inseminated virgin queens and performed MS analysis of the extracted DNA from each spermatheca, as well as queens’ and drones’ tissue. Our results show that median DNA concentration from spermathecas excised prior the storage, regardless of the storing condition and DNA extraction method, were generally lower than median DNA concentration obtained from spermathecas dissected from the whole queens after the storage. Despite the differences in DNA yield from the samples subjected to different storing conditions there was no significant effect of storage method or the DNA extraction method on the amplification success, although fewer samples stored in EtOH amplified successfully in comparison to ATR storing reagent. However, we recommend EtOH as a storing reagent due to its availability, low price, simplicity in usage in the field and in the laboratory, and capability of good preservation of the samples for DNA analysis during transport at room temperature.

## 1 Introduction

Genetic make-up of a honeybee colony is complex because of the polyandrous haplodiploid mating system. The honeybee queen is diploid and mates with several haploid drones. The eggs she lays are either unfertilized and develop into haploid drones or fertilized and develop into diploid workers or queens. The analysis of microsatellite loci in many workers of the same colony allows determination of the genotype of the queen and of the different drones she mated with (Estoup et al., 1995a). Genotyping of the honeybee colony requires planning sample collection well ahead, having in mind the type of data to be acquired. The paternity analysis is not an exception. The paternity of the queen’s offspring is usually determined from known sources through an analysis in which worker brood is sampled (Jensen et al., 2005). Such indirect sampling combined with non-lethal indirect genotyping of the queen may serve in future breeding programs as a genomic information and can provide reliable relationship in the selection process (Châline et al., 2004; Gregory and Rinderer, 2004; Bubnič et al., 2020; Eynard et al., 2022). The drawback of such sampling is that it is costly and time consuming. The alternative is to sacrifice the queen and use it’s spermatheca -- the storage organ of the drones’ semen which provides all the genotypes of the drones that mated with the queen (Estoup et al., 1994; Franck et al., 2002; Carreck et al., 2013).

Regardless of the purpose, the recognized important aspect of handling any biological sample is the assurance of proper transport and storage conditions to adequately preserve the samples for downstream analyses. Degradation of the primary structure of DNA is accelerated by hydrolysis, oxidation and non-enzymatic methylation and is counteracted by DNA repair processes *in vivo* (Lindahl, 1993). With cell death, repair processes, being energy dependent, naturally stop. Moreover, with the collapse of membrane potential and ion channel activity, the osmotic swelling leads to ruptures of membranes thus releasing endogenous nucleases, which in turn lead to immediate DNA degradation. Additionally, lysosome proteases contribute to chromatin breakdown, additionally exposing DNA to nuclease activity. Beside enzymatic DNA degradation, non-enzymatic degradation such as hydrolysis, cross-linkage, and oxidative reactions also plays a role (Alaeddini et al., 2010). Complete loss of long fragments (15–23 kb) was recorded in human forensics and ascribed to autolysis in an interval as short as 0.6–1.5 days in most of the organs (Bär et al., 1988). In some tissues the PCR inhibitors are also released after death, impairing certain polymerase types commonly used in genetic analysis of sampled tissues (Eilert and Foran, 2009).

The DNA amplification success is thus strongly influenced by the preservation method (Straube and Juen, 2013). Preservation of tissue samples is best explored in mammals. Scientific literature, however, offers few insights also in preservation of non-vertebrate tissues. In earthworms freeze-drying of tissue seemed to be preferable, with ethanol storage and simple freezing having yielded between 5% and 15% lower amplification success, respectively (Straube and Juen, 2013).

Field collections of tissue and/or specimen, however, present a collector with a problem of tissue preservation prior to the DNA analysis, for example during the transportation to the laboratory. This is especially important during the longer transport or in cases when the biological material needs to be shipped with limited possibility for sample cooling to prevent the degradation of the tissue and nucleic acids (Eilert and Foran, 2009). An important aspect is also the use of reagents that enable cost efficient and successful subsequent DNA extraction and fragment amplification.

While each biological material has its specific features, many studies explored the suitability of different transport and/or storage conditions of the samples in regards to the sample type and further analyses (Châline et al., 2004; King and Porter, 2004; Zimmermann et al., 2008; Straube and Juen, 2013). Freezing immediately after the sampling would be the most appropriate approach to preserve the DNA (Rissanen et al., 2010) but keeping the samples frozen during the transport may not always be possible or cost efficient. An alternative is to use preservation media to allow storage of the sampled biological material at room temperature. Non-cryogenic tissue preservation successfully addresses DNA quality demands in forensic crime investigation, disaster victim identification (Allen-Hall and McNevin, 2013), research-oriented analysis, or even tissue archives (e.g., biobanking) (Leboeuf et al., 2008).

Use of spermatheca as a source of patriline composition may be preferable compared to the brood samples for various reasons: 1) not all the patrilines are equally represented in the colony at one time point (Brodschneider et al., 2012; Withrow and Tarpy, 2018) thus more consecutive samplings of brood may be required; 2) time-consuming and extensive sampling of the brood may be avoided; 3) in the studies of reproductive interference between honeybee species the offspring might be non-viable and hence missed during sampling (Remnant et al., 2014). The most prominent negative circumstance of usage of such type of tissue is the inevitable sacrifice of the queen.

Few studies have already demonstrated that the honeybee queen’s spermatheca’s content is a suitable yet challenging source for DNA extraction and genotyping of patrilines. However, currently available methods for proper storage of spermatheca and successful extraction of DNA from spermatozoids are only scarcely described (Haberl and Tautz, 1998; Franck et al., 2002; Kahya, 2020). Furthermore, the results of genotyping by microsatellite loci from spermatheca may also be biased due to the errors linked to PCR technique and scoring (Gertsch and Fjerdingstad, 1997). These shortcomings must all be taken into account in the experimental design. In our study we focused on answering two questions: 1) What short-term storage conditions are the most suitable to preserve spermatozoids for subsequent successful DNA extraction and sequencing? 2) Which DNA extraction technique provides suitable results for further research on patriline composition?

Our work gives guidance on collecting, storing and transportation of spermatheca to properly preserve the spermatozoids, as well as the extraction of DNA from the spermatheca’s content.

## 2 Materials and methods

### 2.1 Experimental design

Eighty-eight 1 month old and mated Carniolan honeybee (*Apis mellifera carnica*) queens were obtained from Slovenian breeders. All queens were anesthetized with carbon dioxide prior the storage or dissection. For preservation either Allprotect Tissue Reagent (ATR) (Qiagen) or absolute ethanol were used. Dissection of queens’ spermathecas was performed according to the guidelines under stereomicroscope (Carreck et al., 2013). After dissection, one hind leg per each dissected queen was also stored at −20°C until DNA extraction to obtain pure DNA extract from the queen’s tissue.

**FIGURE 1:**
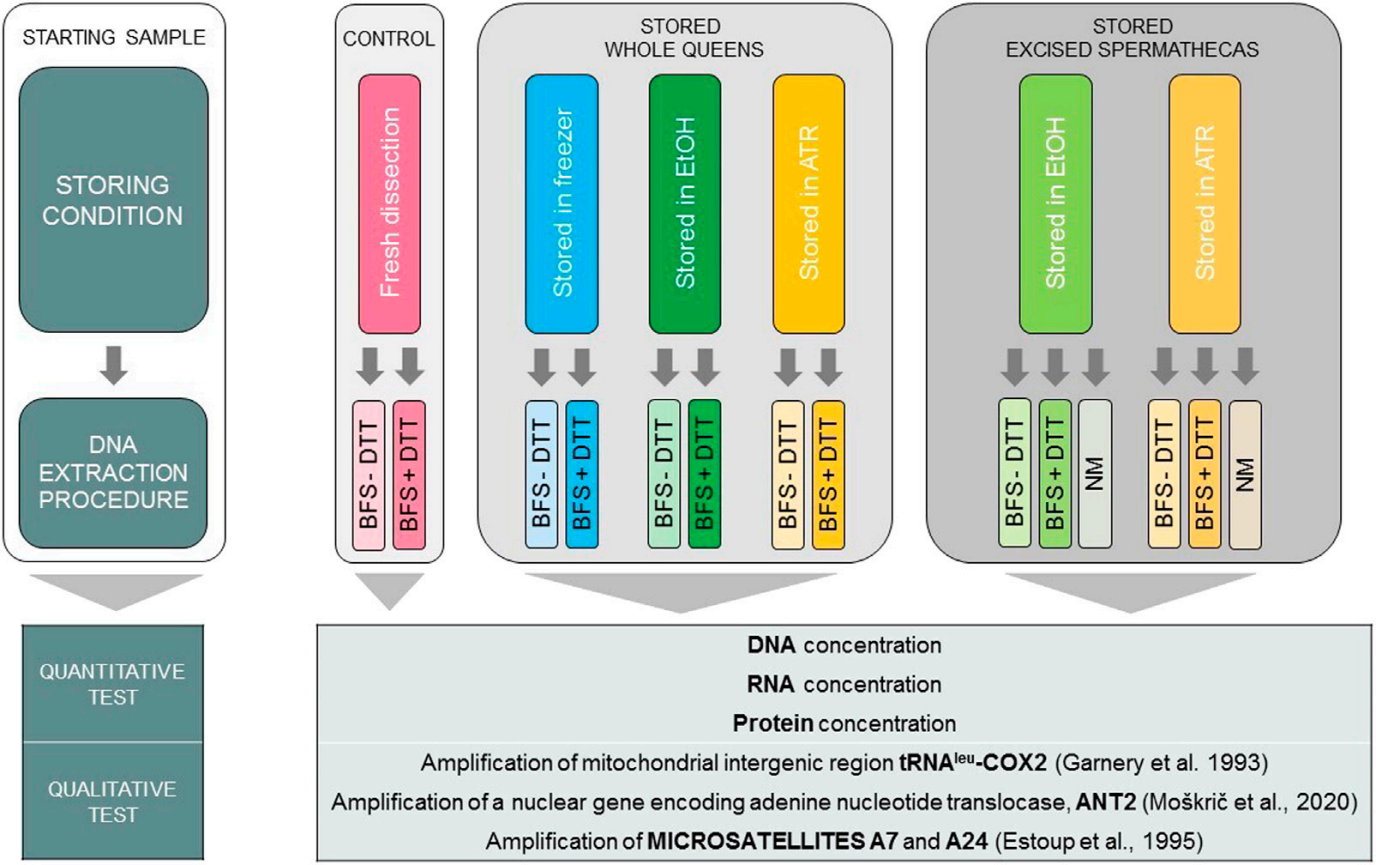
Summarized experimental design of six different storing conditions coupled with three alternative DNA extraction methods. DNA extracts were then tested quantitatively and qualitatively. Abbreviations: ATR, Allprotect Tissue Reagent; EtOH, absolute ethanol; DAF, dissected after freezing; FD, freshly dissected; BFS, QIAamp protocol “Isolation of Total DNA from Body Fluid Stains”; + DTT, with dithiothreitol; −DTT, without dithiothreitol; NM, NucleoMag Tissue Kit’s protocol.

Six different storing conditions of spermathecas were tested: a) whole queens stored in 1.5 mL of Allprotect Tissue Reagent (ATR) at room temperature (*n* = 10), b) excised spermathecas stored in 0.5 mL of ATR at room temperature (*n* = 24), c) whole queens stored in 1.5 mL of absolute ethanol (EtOH) at room temperature (*n* = 10), d) excised spermathecas stored in 0.5 mL of EtOH at room temperature (*n* = 24), e) whole queens frozen at −20°C (DAF, dissected after freezing; *n* = 10) and f) fresh whole queens (freshly dissected, FD; *n* = 10). Except for the fresh queens as starting material, all the samples were exposed to storing conditions listed above for 6 to 8 days. In this way we simulated the conditions during transportation of the samples at room temperature, without the possibility of cooling the samples. Storing conditions of spermathecas and subsequent DNA extraction methods are summarized in Figure 1.

### 2.2 DNA extraction methods

Complementing six storing conditions, two triflingly different DNA extraction protocols based on QIAamp protocol “Isolation of Total DNA from Body Fluid Stains” (hereafter BFS) (QIAamp, 2020) from DNA Investigator Handbook 2020 were tested, differing only in extraction with and without added dithiothreitol (DTT). Additionally, the third method of semi-automated extraction using NucleoMag Tissue Kit (Macherey-Nagel) was used in extraction of DNA from spermathecas in ATR and EtOH.

More in detail, samples which were stored as whole queens and fresh whole queens were dissected just prior the DNA extraction. Excised spermathecas were transferred to 1.5 mL Eppendorf tubes to which 300 µL ATL buffer and 20 µL proteinase K were added. Spermathecas were pop-opened by gentle squeezing with tweezers to release the spermatozoids. The membrane of spermatheca was then removed although tiny membrane pieces may have remained, so we assumed possible contamination of the extract with queen’s DNA. 20 μL DTT (1 M) was added to selected portion of the samples; all samples (with and without DTT added) were then incubated at 56°C and 500 rpm for 1 h 300 μL AL buffer was added followed by another 10 min incubation at 70°C and 500 rpm. Next, 150 µL of absolute ethanol was added followed by vortexing. The samples were then transferred to QIAamp MinElute columns with collection tubes and centrifuged on 8,000 rpm for 1 min. Washing was continued with 500 µL AW1 buffer, following by 700 µL AW2 buffer and 700 µL absolute ethanol followed by centrifugation at 14.000 rpm for 3 min and drying at 56°C for 3 min. For the elution 50 µL AE buffer was added and the samples were incubated for 1 min at room temperature. Finally, the samples were centrifuged at 14.000 rpm for 1 min and stored at −20°C.

DNA extractions from selected samples were carried out with NucleoMag Tissue Kit and its slightly modified protocol (Genomic DNA from tissue—User manual NucleoMag^®^ Tissue) with The MagMAX Express-96 Deep Well Magnetic Particle Processor (Thermo Fisher Scientific). Spermathecas were moved to 1.5 mL Eppendorf tubes which contained 100 µL T1 lysis buffer and 10 µL proteinase K. The spermathecas were pop-opened similarly as described above. Samples were incubated overnight at 56°C. Next day the samples were centrifuged at 5.600 × g for 5 min and Flex 96 Standard Plates (Thermo Fisher Scientific) were prepared. To row A 110 µL binding buffer MB2, 10 µL NucleoMag B-Beads and 90 µL samples ware added. To row B 150 µL wash buffer BM3, to row C 150 µL wash buffer BM4, to row D 200 µL wash buffer BM5 and to row E 50 µL elution buffer MB6 were added. The plates were then transferred to MagMAX Processor and the program was set as described in the Manufacturer’s protocol. Additionally, one hind leg per queen was used for DNA extraction to obtain the DNA from each queen. The legs were transferred to 1.5 mL Eppendorf tubes with T1 lysis buffer and proteinase K and crushed with mini pestles, vortexed and incubated overnight. DNA extraction was then carried out with the MagMAX Express-96 Deep Well Magnetic Particle Processor (Thermo Fisher Scientific) and NucleoMag Tissue Kit (Macherey-Nagel) as described above. All the samples of extracted DNA were stored at −20°C until further use.

### 2.3 Quantitative check of the DNA extracts

DNA, RNA, and protein concentrations in DNA extracts were measured using Qubit fluorometer (Thermo Fisher Scientific) and the corresponding kits: Qubit dsDNA HS (High Sensitivity) Assay Kit, RNA HS Assay Kit and Protein Assay Kit (all Thermo Fisher Scientific) following the manufacturer’s protocols. The results were statistically analyzed and visualized in R studio (for more details see Supplementary Material). For multiple pairwise comparisons between combinations of tested storage conditions and isolation methods for DNA concentrations a non-parametric Kruskal-Wallis test with Conver-Iman *post hoc* test were chosen for statistical analysis, since non normally distributed data was obtained.

### 2.4 Qualitative check of the DNA extracts by PCR amplification and sequencing

Two sets of primers for amplification of genetic markers were selected. First set of primers (ANT2) amplifies a part of a nuclear ANT gene, encoding adenine nucleotide translocase which is embedded in the mitochondrial membrane (Kim et al., 2010). ANTapF2 and ANTapR2 oligonucleotide primers were used. The amplified region is located in between two intronic regions and is approximately 770 bp-long (Moškrič et al., 2020). The PCR amplification was carried out using DreamTaq PCR Master Mix (Thermo Fisher Scientific). The reaction mixture consisted of 7.5 µL of 2x DreamTaq MasterMix buffer, 0.2 µL of each oligonucleotide primer (20 µM), 2 µL of extracted DNA and ddH_2_O up to the final volume of 15 µL. The conditions of amplification were as follows: initial denaturation step of 3 min at 94°C was followed by 35 cycles of denaturation step of 1 min at 94°C, annealing step of 1 min at 59°C and extension step of 1 min at 72°C. Final extension step lasted for 5 min at 72°C. The second set of primers (E2 and H2) amplifies a mitochondrial intergenic region tRNA^leu^-COX2, which is 563–1,011 bp long (Garnery et al., 1992, 1993). The PCR amplification mixture was the same as described above. The conditions of amplification were as follows: initial denaturation step of 3 min at 95°C was followed by 40 cycles of denaturation step of 1 min at 95°C, annealing step of 1 min at 50°C and extension step of 1 min at 72°C. Final extension step lasted for 5 min at 72°C.

Amplified PCR products were visualized using 1% agarose gel electrophoresis in 0.5x TBE buffer with 1.3 µL ethidium bromide. 1 μL 6x TriTrack DNA Loading Dye (Thermo Fisher Scientific) was added to 5 µL PCR products before loading. GeneRuler 100 bp Plus DNA Ladder (Thermo Fisher Scientific) was used for standard. The electrophoresis was run at 100 V for 35 min. PCR product were visualized with GeneGenius Bio Imaging System (Syngene).

ANT2 and tRNA^leu^-COX2 PCR products from two samples per each storing condition and BFS extraction method were cleaned with ExoSAP-IT PCR Product Cleanup Reagent (Applied Biosystems) following the manufacturer’s protocol. The cleaned-up PCR products were sent to SEQme (Czech Republic) for sequence determination *via* Sanger sequencing using both sequencing primers. Chromatograms were assembled and edited using Geneious Prime (https://www.geneious.com). Edited sequences for each marker separately were aligned using Mafft plug-in (Katoh and Standley, 2013). Sequences were deposited in GenBank repository (NCBI).

### 2.5 Quality check by genotyping by microsatellites and patriline analysis

To determine whether the extracted DNA is suitable also for genotyping by microsatellite reads (MS), 68 DNA extracts from spermathecas were used for amplification of the microsatellite loci. For the naturally mated queens the microsatellites A7 and A24 were used. The instrumentally inseminated queens were tested on microsatellites A7 and A113 (Table 1). The PCR was carried out using Type-it Microsatellite PCR Kit (Qiagen). The reaction mixture consisted of 6.75 µL of 2x Type-It MasterMix, 1.35 µL of Q solution, 0,15 µL of each oligonucleotide primer (0.2 µM), 3 µL of extracted DNA and ddH_2_O up to the final volume of 15 µL. The conditions of amplification were as follows: initial denaturation step of 5 min at 95°C was followed by 35 cycles of denaturation step of 1 min at 94°C, annealing step of 1 min 30 s and temperature from 58 to 52°C and extension step of 1 min at 72°C. Every 5th cycle the annealing temperature was increased for 1°C. Final extension step lasted for 5 min at 72°C.

**TABLE 1:** Microsatellite loci used in this study.

Microsatellite	Forward primer	Reverse primer	Ta (°C)	Fragment size	Reference
A7	5′-GTT​AGT​GCC​CTC​CTC​TTG​C-3′	5′-CCC​TTC​CTC​TTT​CAT​CTT​CC-3′	58	83.8–148	Estoup et al. (1995b)
A24	5′-CAC​AAG​TTC​CAA​CAA​TGC-3′	5′-CAC​ATT​GAG-GAT​GAG​CG-3′	55	89.98–107.96	Estoup et al. (1995b)
A113	5′-CTC​GAA​TCG​TGG​CGT​CC-3′	5′-CCT​GTA​TTT​TGC​AAC​CTC​GC-3′	60	193,06–238	Estoup et al. (1995b)

Horizontal gel electrophoresis using 1.8% agarose gel in 0.5x TBE buffer with ethidium bromide was performed. 1 μL 6x TriTrack DNA Loading Dye (ThermoFisherScientific) was added to 5 µL PCR products before they were transferred to the gel. GeneRuler 100 bp Plus DNA Ladder (Thermo Fisher Scientific) was used as standard. The electrophoresis was run at 100 V for 45 min. PCR products were visualized with GeneGenius Bio Imaging System (Syngene).

Successfully amplified samples were then prepared for capillary electrophoresis. Each well of 96-well plate was filled with 10 µL formamide, 0.35 µL size standard LIZ500 and 1.3 µL of amplified DNA. The samples were denaturated at 95°C for 2 min and then for 2 min incubated on ice. Microsatellite analysis was performed on 3500 Genetic Analyzer for Fragment Analysis (Applied Biosystems). The traces were visualised and peak calling was performed using the microsatellite plugin in Geneious Prime.

Lastly, to test the usefulness of stored spermatheca as a source of patriline composition, three virgin queens were instrumentally inseminated. Instrumental insemination was performed following the standard protocol (Cobey et al., 2013). Each queen was inseminated by several drones (15, 11, 11). For each drone hind legs were stored at −20°C until DNA extraction. After the insemination, queens were kept alive for additional 3 days, then were dissected to obtain the spermathecas. For each queen hind legs were stored. Next, DNA extraction of spermatozoids from spermathecas was performed using the protocol BFS with DTT. DNA extraction of legs from queens and drones was carried out with NucleoMag Tissue Kit. For genotyping by microsatellite reads, two microsatellite loci were selected for amplification (Table 1): A7 and A113. The PCR was carried out using Type-it Microsatellite PCR Kit (Qiagen). The extractions of DNA as well as the following procedure from the reaction mixture to the capillary electrophoresis and microsatellites analysis were the same as described above.

## 3 Results

To evaluate which combination of storage conditions and DNA extraction method is the most suitable, we performed quantitative as well as qualitative tests of the DNA extracts. The quantitative test included determining the concentration of DNA, as well as the presence of RNA and proteins in the extracts. The qualitative test included the amplification of two genetic markers (ANT2 and COI) by PCR. Last step was the test whether the quality of the extracted DNA allows successful MS genotyping to assess the patriline composition.

### 3.1 Quantity of DNA, RNA and proteins in extracts

**FIGURE 2:**
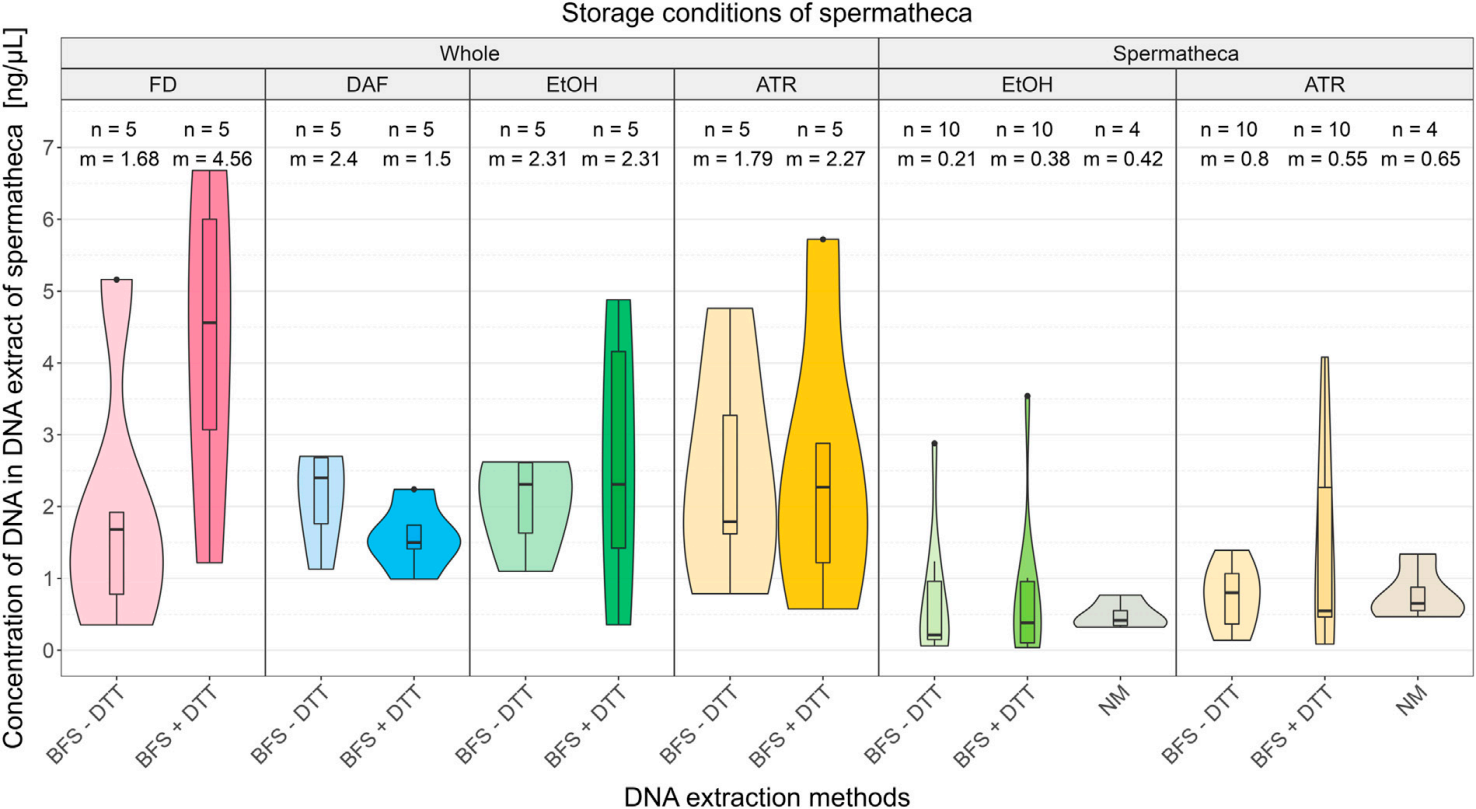
Comparison of DNA concentration in DNA extracts obtained from different storing conditions of honeybee samples and DNA extraction method with or without DTT from spermathecas. Abbreviations: ATR, Allprotect Tissue Reagent; EtOH, absolute ethanol; DAF, dissected after freezing; FD, freshly dissected; BFS, QIAamp protocol “Isolation of Total DNA from Body Fluid Stains”; + DTT, with dithiothreitol; −DTT, without dithiothreitol; NM, NucleoMag Tissue Kit’s protocol; n, sample size; m, median.

Results of comparison of different storing conditions and DNA extraction methods are shown in Figure 2 (all DNA concentrations are presented in Supplementary Material Chapter S1). The results from the statistical analysis for a non-parametric Kruskal-Wallis test with Conver-Iman *post hoc* test are given in Supplementary Material Chapter S2.

Median DNA concentrations obtained from spermathecas excised prior the storage, regardless the storing condition and DNA extraction method, were lower than median DNA concentrations obtained from spermathecas dissected from the whole queens after the storage (range of medians 0.21 ng/μL—0.80 ng/μL vs. 1.50 ng/μL - 4.56 ng/μL).

When comparing values between storing conditions within »whole queen« group the median obtained DNA concentration was higher in EtOH compared to ATR: 2.31 ng/μL vs. 1.79 ng/μL (BFS - DTT) and 2.31 ng/μL vs. 2.27 ng/μL (BFS + DTT) regardless of the extraction methods. Furthermore, within »whole queen« group the obtained DNA concentrations were higher with DNA extraction method BFS with DTT in two out of four storing conditions (FD and ATR; 4.56 ng/μL vs. 1.68 ng/μL; 2.88 ng/μL vs. 1.79 ng/μL). Storing in EtOH resulted in equal median DNA concentrations (2.31 ng/μL). In case of queens that were frozen before dissection, the situation was reversed: use of DTT decreased the median concentration of extracted DNA (1.5 ng/μL vs. 2.4 ng/μL). Overall, the highest DNA concentrations were obtained from fresh whole queens using DNA extraction method BFS with DTT. For statistical significance of pairwise multiple comparisons between results of all storing methods and DNA extraction methods see Supplementary Table S2.2.1.

RNA was not detected in any of DNA extracts. While proteins were detected in 9 out of 88 DNA extracts, namely in all the extracts in which the spermathecas were directly stored in EtOH or ATR and the NM method for DNA extraction was used, and in one extract where the spermatheca was stored directly in ATR and the DNA was extracted with the BFS—DTT method. NM extraction method resulted in overall the highest concentration of proteins in the extracts. The lowest protein concentration (141 ng/μL) was measured in DNA extract from the spermatheca stored directly in ATR and the DNA extracted with the BFS—DTT. Average protein concentration where EtOH with NM method was used was 218 ng/μL (between 170 and 283 ng/μL) and 299 ng/μL (between 279 and 319 ng/μL) where ATR with NM method was used.

### 3.2 Qualitative check of the DNA extracts

In only three cases out of 88 tested samples, we were not able to detect visible fragments on agarose gel when amplifying mitochondrial tRNA^leu^-COX2 marker: once from spermatheca stored in ATR with MN extraction (1 of 4), once from spermatheca stored in EtOH with BFS—DTT extraction (1 of 10) and once from whole queen stored in EtOH with BFS—DTT extraction (1 of 5). Similarly, the nuclear ANT2 marker was successfully amplified in 81 out of 88 cases. Amplification failed in one case of ATR-stored spermatheca with BFS-DTT extraction, two cases of EtOH-stored spermatheca with BFS-DTT extraction, two cases of EtOH-stored spermatheca with BFS + DTT extraction, one case when extracting DNA from EtOH-whole-queen spermatheca with BFS + DTT extraction and once amplification failed in BFS-DTT extracted from EtOH-stored whole queen. The resulting figures of the gel electrophoresis and table are presented in Supplementary Material Chapter S3.

ANT and tRNA^leu^-COX2 PCR products from two samples per each storing condition and BFS extraction method were sequenced in both directions and chromatograms obtained were assembled using Geneious Prime. For tRNA^leu^-COX2 products all sequences (24 out of 24) were readable. Four different haplotypes were determined and the sequences are available in GenBank repository (NCBI) under accession numbers OQ383216—OQ383219. The clear sequence reads were obtained for 22 ANT PCR products out of 24 sequences. Two sequences of ANT fragment that were of poor quality originated from 1) spermatheca stored in EtOH with BFS + DTT DNA extraction and 2) spermatheca stored in ATR with BFS–DTT DNA extraction, respectively. In both cases reliable sequence determination from the chromatograms was not possible. The sequence of the amplified fragment was the same in all reads and is available in GenBank repository under accession number OQ411004.

Regardless of the storage and DNA extraction conditions, the DNA fragments were long enough to support successful amplification of ANT2 (approximately 770 bp in length) and tRNA^leu^-COX2 (approximately 550 bp in length) regions in most of the cases. In groups FD and DAF, the success was 100%; the “Whole” group performed worser, where in samples from EtOH-stored queens and spermathecas success of amplification dropped to 80%–90% for BFS - DTT extraction and 80% for ANT2 in BFS + DTT extraction. ATR supported PCR of reasonable quality for both selected DNA fragments. In stored spermathecas, situation was similar in EtOH.

### 3.3 Suitability of transport method for use in microsatellite analysis

**FIGURE 3:**
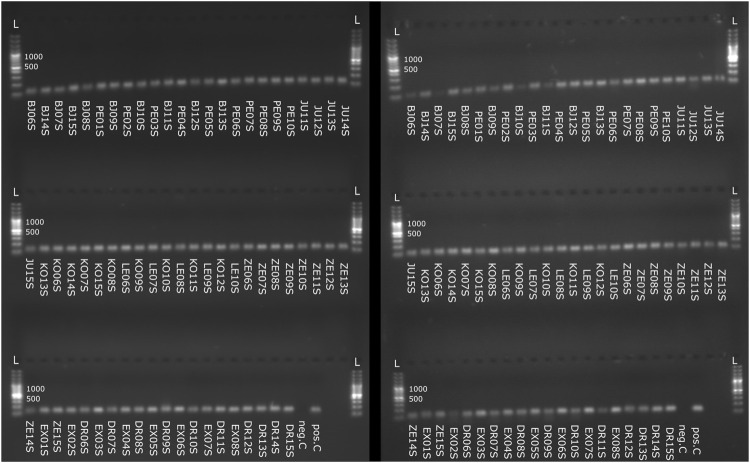
Agarose gel electrophoresis of amplified microsatellite loci from spermatheca samples. A07 microsatellite locus is presented on the left, A24 microsatellite locus is presented on right. L—100 bp standard ladder, neg.C—Negative control, pos.C—Positive control.

To verify the value of stored spermatheca as a source of patriline composition, the amplification of selected microsatellites was carried out from DNA extracts from spermathecas and the corresponding queens’ legs. Two microsatellite loci were tested, A07 and A24. We were able to perform the readouts of microsatellite peaks from all the DNA extracts from spermathecas involved in this trial (68/68), regardless of the storage method or extraction type. Microsatellite loci amplification success is presented in Figure 3 and in Supplementary Material Chapter S4.

Two to seven alleles of A07 microsatellite were detected in a spermatheca. On average, 4.4 alleles were detected in each spermatheca. All queen samples except one were successfully genotyped and all were of heterozygous genotype. A single allele of the queen was confirmed in 23 spermathecas while both alleles corresponding to the queen were found in 44 spermathecas. For A24 microsatellite all queens were of heterozygous genotype. Up to four alleles of A24 microsatellite were detected in spermatheca. On average 2.6 alleles were detected in each spermatheca. A single allele corresponding to the queen’s genotype was confirmed in 27 spermathecas. Both alleles from the queen were confirmed in 41 spermathecas.

**FIGURE 4:**
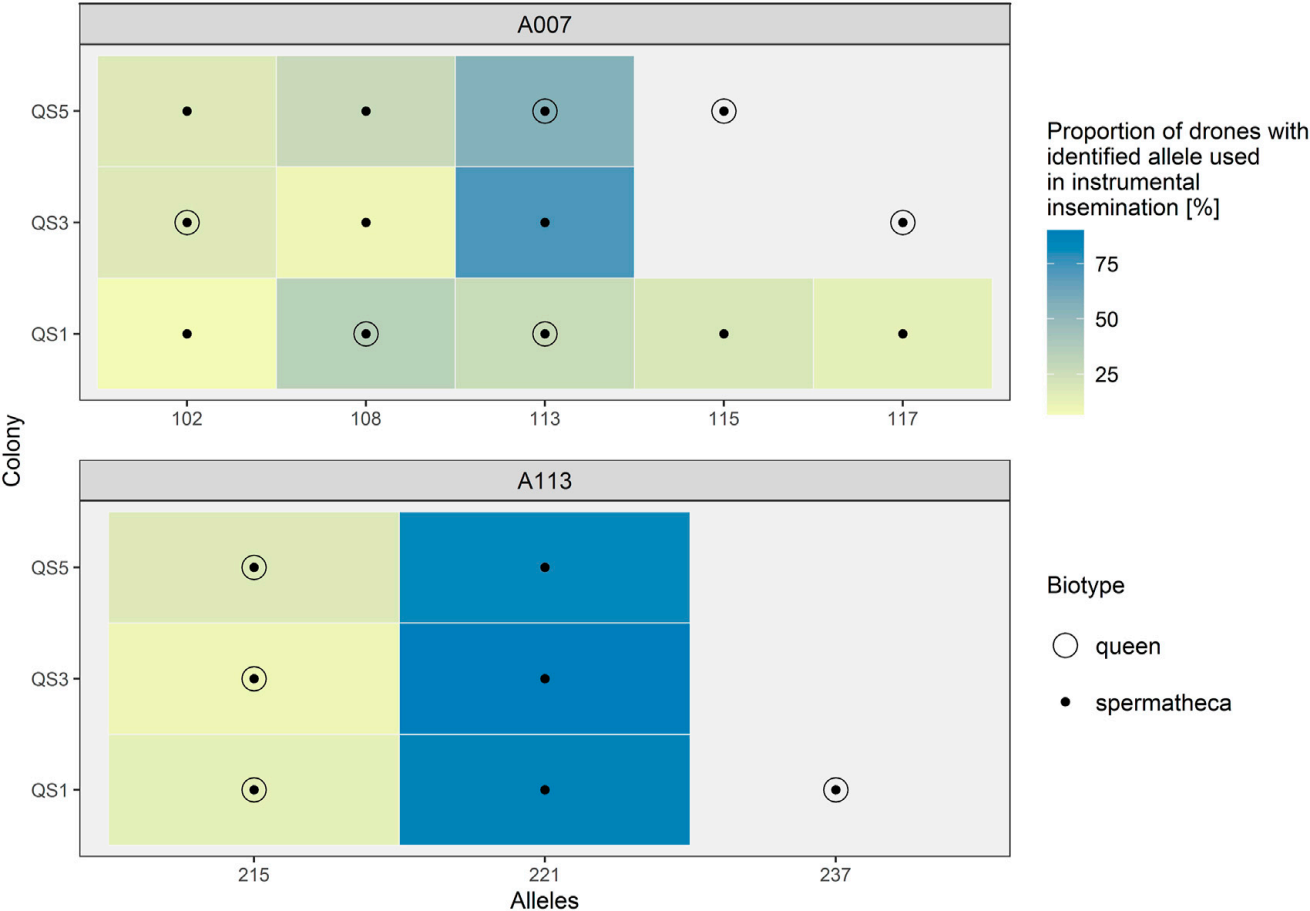
Genotypes of A7 and A113 microsatellite loci of spermathecas from instrumentally inseminated queens, their corresponding queens and drones. Legend: The genotypes of drones are presented as coloured rectangles—The colour indicates the proportion of detected allelic variants in drones. Black dots represent the genotypes detected in each spermatheca. Empty circles indicate the genotype of the queen.

To additionally illuminate usefulness of storage methods with focus on patriline composition, we have instrumentally inseminated three virgin queens by several drones (15, 11, 11). For genotyping of spermatozoa from spermatheca, amplification of selected microsatellites was carried out from spermatheca, drones that were used for insemination and the corresponding queens’ legs. Two microsatellites were used, A07 and A113 (Table 1). The results of genotyping are shown in Figure 4 (gel electrophoresis figures of amplification success are presented in Supplementary Material Chapter S5). For both microsatellite loci we were able to successfully determine all alleles that were identified in drones, used in insemination of that particular queen. As expected, we were also able to determine the alleles of the queens in the DNA extract from corresponding spermatheca. In Figure 4 the empty circles represent the genotype of the queen.

## 4 Discussion

The complex nature of reproductive biology of honeybees demands adjustments in sampling and genotyping in comparison to other managed animals. To explore the genotype of the colony, a large number of worker individuals, preferably in late pupal stages, must be collected (Jensen et al., 2005; Evans et al., 2013). This approach has been widely used and is considered the most appropriate to obtain the data on patriline number and composition (Franck et al., 1999; Franck et al., 2002; Jensen et al., 2005; Brodschneider et al., 2012; Withrow and Tarpy, 2018). However, this kind of sampling coupled with downstream molecular analyses is costly and time-consuming. Importantly, it does not provide answers related to patriline composition of the colony prior the mated or inseminated queen forms a colony which prolongs and delays the research for at least up to one season. Therefore, more straight forward, yet destructive alternative, is to sample the queens soon after mating flights are completed and patriline information is extracted directly from the mixture of spermatozoids from their spermathecas. The main purpose of this study was to investigate methods of short-term storage that would allow proper preservation, transport and retrieval of genetic information contained in honeybee queen’s spermatheca.

Our results show that median DNA concentrations from spermathecas excised prior the storage, regardless the storing condition and DNA extraction method, were generally lower than median DNA concentrations obtained from spermathecas dissected from the whole queens after the storage. Spermatheca excised prior the storage thus may be less reliable type of biological sample for further molecular analyses—storing of intact honeybee queen is recommended.

For preservation of the samples at room temperature either Allprotect Tissue Reagent (ATR) (Qiagen) or absolute ethanol (EtOH) were used. The selection of those two reagents was based on the fact that both are supposed to successfully preserve various tissues for further molecular analyses. The all-weather ethanol is reviewed often, and the experience is most diverse, its preservation efficiency depending on the tissue. ATR is one of several proprietary preservatives on the market. Despite the differences in DNA yield from the samples subjected to different storing conditions there was no significant effect of storage method or the DNA extraction method on the amplification success, although fewer samples stored in EtOH amplified successfully in comparison to ATR storing reagent (Supplementary Material). Nevertheless, absolute EtOH is still recommended as a storing reagent due to its availability, low price, simplicity in usage in the field and in the laboratory, and capability of good preservation of the samples during transport (shipment) at room temperature. On the other hand, as described by the manufacturer, ATR provides immediate stabilization of DNA, RNA, and proteins in tissue samples at room temperature thus allowing reliable downstream analysis. In practice, our results show only one failed DNA amplification (spermatheca in ATR, ANT2 marker) regardless of extraction mode when using ATR. Thus, ATR proved to be an excellent storing reagent with multipurpose usage and its main drawback seems to be the high price. In some reviews, ATR is sorted in the same category as RNALater (Ambion), which is also pricey (Nagy, 2010).

Different tissue types yield different amount and quality of extracted DNA (Lopez Vaamonde et al., 2012; Bubnič et al., 2020). In our work, the median quantities of DNA were between 0.21 and 4.56 ng/μL. The results of the obtained DNA quantities could be divided roughly into two groups, with median DNA quantities being above and below 1 ng/μL. The two groups correspond to two storage methods: either whole queen or excised spermatheca. As expected, DNA extractions from spermatheca of freshly dissected queens resulted in the highest DNA concentrations, which were best when combined with extraction method using DTT. Frozen samples (group DAF) also yielded a comparable DNA quantity, only this time median was higher in a group where DNA was extracted without DTT.

Spermatheca is s spherical sac in which the spermatozoa are stored and kept alive for the life of the queen (Carreck et al., 2013). It is minute in size, with calculated volume of approximately 0.5 up to 1.75 mm^3^ (Prešern and Smodiš Škerl, 2019). Also the quantity of spermatozoids inside the spermatheca is variable, estimated from 1 up to 10 million (Collins and Pettis, 2012; Baer et al., 2016; Kovačić et al., 2016; Prešern and Smodiš Škerl, 2019). The variability of size and the quantity of spermatozoids may also be a likely reason for the variability of the measured DNA yield in our study.

The measured concentrations of DNA differed between methods of storage and extraction, yet the differences seem to be small enough not to be reflected in the second stage quality tests. Nuclear (ANT2) and mitochondrial (region tRNA^Leu^—COX2) markers are useful in taxonomy, phylogeny and population studies (Teske and Beheregaray, 2009; Evans et al., 2013). Furthermore, the successful amplification of DNA fragments is an indicator of low fragmentation of DNA, which is indirectly connected with the quality of extracted DNA. More than 91% success in amplification of ANT2 fragment, which is approximately 770 bp long, hints that the extracted DNA is of reasonable quality.

In our work, extractions using DTT performed slightly better compared to BFS-DTT when amplifying tRNA^leu^—COX2 marker. DTT is a strong reducing agent which breaks down disulfide bonds in proteins and is often used in forensic DNA analysis to enhance the yield of DNA extraction from sperm (Yoshida et al., 1995; Hudson et al., 2020). Whether such slight improvement of yield justifies theuse of DTT depends mostly on funds available and on available laboratory equipment, since DTT requires handling in fume hood.

Some of the DNA extracts from spermathecas included at least one microsatellite variant identical to the variant that was also identified in the corresponding queen. This is either because the drone carries the same variant on that locus as the queen or the presence of this variant in the DNA extract of spermatheca is due to the contamination of queen’s DNA during the preparation of the spermatheca for DNA extraction.

We confirmed the presence of queen’s DNA in spermathecal content in the three cases of virgin queens after artificial insemination with known drones, whose legs were collected for identification of their own microsatellite variants (Figure 4). Thus, the DNA extraction procedure of spermathecal content needs to be further optimised to allow the entire removal of the spermathecal sac prior the lysis to prevent the carry-over of queen’s DNA.

The DNA content of spermatheca may be especially useful source of genotyping information for honeybee breeding and selection purposes. One of the key elements, important in establishment of genetic gain in honeybees, is successful mating control. However mating control in honeybees is difficult to establish and is often missing (Uzunov et al., 2022). The evaluation of mating control success and genomic selection may be achieved through genotyping of brood of successfully mated queens (Jensen et al., 2005; Jones et al., 2020). As explained above, the downsides of such time-consuming, extensive and demanding sampling may be overcome by specialized genotyping procedures in which the content of spermatheca would be successfully used as a single sample representing the genotype of the entire colony.

Our work expands the experience with simple short-term storage of tissues in invertebrates for DNA analyses, hopefully providing evidence of reduced need for more expensive agents. Furthermore, the diverse power of DNA extraction techniques success in invertebrates is explored, which widens the possibility of obtaining DNA material of proper quality and quantity for downstream analyses.

## Data Availability

The datasets presented in this study can be found in online repositories. The names of the repository/repositories and accession number(s) can be found below: https://www.ncbi.nlm.nih.gov/genbank/, OQ383216 https://www.ncbi.nlm.nih.gov/genbank/, OQ383217 https://www.ncbi.nlm.nih.gov/genbank/, OQ383218 https://www.ncbi.nlm.nih.gov/genbank/, OQ383219 https://www.ncbi.nlm.nih.gov/genbank/, OQ411004.
