# Supplementary material for: Cutting corners: The impact of storage and DNA extraction on quality and quantity of DNA in honeybee (Apis mellifera) spermatheca
Source: Front Physiol. 2023 Mar 3;14:1139269. doi: 10.3389/fphys.2023.1139269 (PMC10020693; doi:10.3389/fphys.2023.1139269)
Supplement: Supplementary file 1 [file DataSheet1.docx]

Supplementary Material

# DNA concentrations in DNA extracts

**Supplementary Table 1.1.** DNA concentration in DNA extracts obtained from different storing conditions of honeybee samples and DNA extraction method with or without DTT from spermathecas. Abbreviations: ATR - Allprotect Tissue Reagent, EtOH - absolute ethanol, DAF - dissected after freezing, DF - freshly dissected, BFS - QIAamp protocol 'Isolation of Total DNA from Body Fluid Stains', + DTT - with dithiothreitol, - DTT - without dithiothreitol, NM - NucleoMag Tissue Kit's protocol

| **Storing method** | | **DNA isolation method** | **DNA concentration (ng/µL)** | **Sample** |
| --- | --- | --- | --- | --- |
| Spermatheca | ATR | BFS - DTT | 0.908 | ME7 |
|  |  |  | 1.390 | ME8 |
|  |  |  | 0.139 | ME9 |
|  |  |  | 0.218 | ME10 |
|  |  |  | 0.300 | ZE12 |
|  |  |  | 0.912 | ZE13 |
|  |  |  | 1.120 | ZE14 |
|  |  |  | 0.564 | ZE15 |
|  |  |  | 0.696 | ME6 |
|  |  |  | 1.160 | ZE11 |
|  |  | BFS + DTT | 4.080 | LE12 |
|  |  |  | 0.580 | LE13 |
|  |  |  | 0.512 | LE14 |
|  |  |  | 3.060 | LE15 |
|  |  |  | 0.086 | BJ7 |
|  |  |  | 0.230 | BJ8 |
|  |  |  | 0.516 | BJ9 |
|  |  |  | 0.448 | BJ10 |
|  |  |  | 2.160 | LE11 |
|  |  |  | 2.300 | BJ6 |
|  |  | NM | 0.724 | EX6 |
|  |  |  | 1.340 | EX7 |
|  |  |  | 0.468 | EX8 |
|  |  |  | 0.580 | EX5 |

|  | EtOH | BFS - DTT | 1.240 | JU7 |
| --- | --- | --- | --- | --- |
|  |  |  | 2.880 | JU8 |
|  |  |  | 1.070 | JU9 |
|  |  |  | 0.158 | JU10 |
|  |  |  | 0.240 | DR7 |
|  |  |  | 0.147 | DR8 |
|  |  |  | 0.189 | DR9 |
|  |  |  | 0.120 | DR10 |
|  |  |  | 0.632 | JU6 |
|  |  |  | 0.061 | DR6 |
|  |  | BFS + DTT | 3.540 | ME11 |
|  |  |  | 0.544 | ZE6 |
|  |  |  | 0.058 | ME12 |
|  |  |  | 0.213 | ME13 |
|  |  |  | 0.066 | ME14 |
|  |  |  | 1.010 | ME15 |
|  |  |  | 0.221 | ZE7 |
|  |  |  | 0.972 | ZE8 |
|  |  |  | 0.916 | ZE9 |
|  |  |  | 0.036 | ZE10 |
|  |  | NM | 0.352 | EX2 |
|  |  |  | 0.768 | EX3 |
|  |  |  | 0.322 | EX4 |
|  |  |  | 0.480 | EX1 |
| Whole queen | ATR | BFS - DTT | 0.788 | LE7 |
|  |  |  | 4.760 | LE8 |
|  |  |  | 1.790 | LE9 |
|  |  |  | 1.620 | LE10 |
|  |  |  | 3.270 | LE6 |
|  |  | BFS + DTT | 0.576 | KO12 |
|  |  |  | 5.720 | KO13 |
|  |  |  | 2.270 | KO14 |
|  |  |  | 1.220 | KO15 |
|  |  |  | 2.880 | KO11 |
|  | EtOH | BFS - DTT | 1.100 | KO7 |
|  |  |  | 2.620 | KO8 |
|  |  |  | 1.630 | KO9 |
|  |  |  | 2.310 | KO10 |
|  |  |  | 2.610 | KO6 |

|  |  | BFS + DTT | 0.355 | JU12 |
| --- | --- | --- | --- | --- |
|  |  |  | 4.880 | JU13 |
|  |  |  | 1.420 | JU14 |
|  |  |  | 2.310 | JU15 |
|  |  |  | 4.160 | JU11 |
|  | DAF | BFS - DTT | 1.760 | BJ12 |
|  |  |  | 1.130 | BJ13 |
|  |  |  | 2.700 | BJ14 |
|  |  |  | 2.680 | BJ15 |
|  |  |  | 2.400 | BJ11 |
|  |  | BFS + DTT | 2.240 | DR11 |
|  |  |  | 1.500 | DR12 |
|  |  |  | 0.992 | DR13 |
|  |  |  | 1.410 | DR14 |
|  |  |  | 1.740 | DR15 |
|  | FD | BFS - DTT | 5.160 | PE7 |
|  |  |  | 1.680 | PE8 |
|  |  |  | 0.780 | PE9 |
|  |  |  | 1.920 | PE10 |
|  |  |  | 0.354 | PE6 |
|  |  | BFS + DTT | 3.070 | PE2 |
|  |  |  | 6.000 | PE3 |
|  |  |  | 1.220 | PE4 |
|  |  |  | 6.680 | PE5 |
|  |  |  | 4.560 | PE1 |

# Multiple comparisons of DNA concentrations between of all storing methods and DNA extraction methods

## Assumption tests of normality

Test of normality was carried out with Q-Q plot (function ggqqplot from the package ggpubr) and Shapiro–Wilk test (function shapiro.test from the package stats), which null hypothesis states that the sample comes from a normally distribution population in other words data from each combination is normally distributed. Combinations with p-values less than 0.05 are not normally distributed. Below the Q-Q plots (Supplementary Figures 1.1.) and the table with p-values of Shapiro–Wilk test (Supplementary Table 1.1.) for each combination is shown. Only three combination (ATR, Spermatheca| BFS + DTT, EtOH, Spermatheca | BFS + DTT, EtOH, Spermatheca | BFS - DTT) had p-values lower than 0.05.


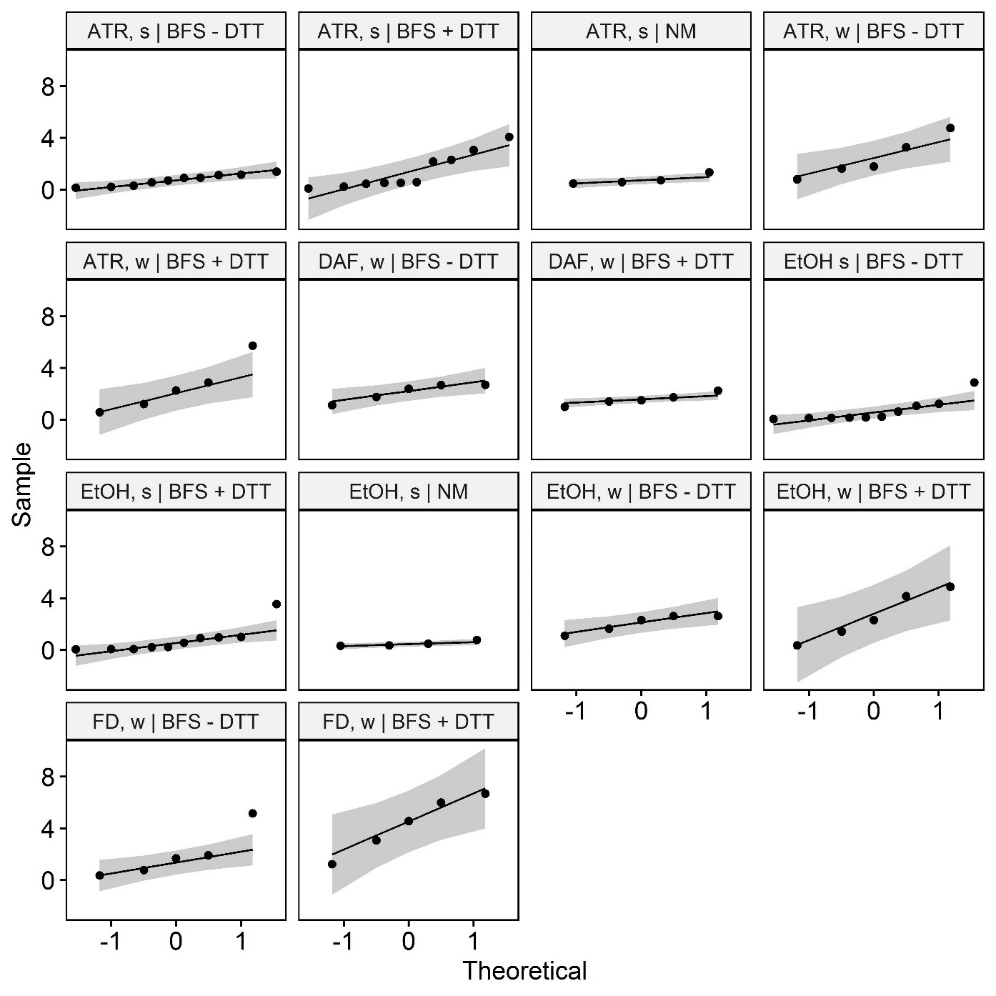


Supplementary Figures 2.1.1. Q-Q plots for test of normality. Abbreviations: ATR - Allprotect Tissue Reagent, EtOH - absolute ethanol, DAF - dissected after freezing, DF - freshly dissected, BFS - QIAamp protocol 'Isolation of Total DNA from Body Fluid Stains', + DTT - with dithiothreitol, - DTT without dithiothreitol, NM - NucleoMag Tissue Kit's protocol, s - spermatheca, w - whole queen

**Supplementary Table 2.1.2.** Test statistic W and p-values of Shapiro–Wilk test

| **Storing condition and DNA isolation method** | **W** | **p.values** |
| --- | --- | --- |
| EtOH, w \| BFS + DTT | 0.9516 | 0.7483 |
| EtOH, w \| BFS - DTT | 0.8709 | 0.2701 |
| ATR, w \| BFS + DTT | 0.9175 | 0.5141 |
| ATR, w \| BFS - DTT | 0.9328 | 0.6159 |
| ATR, s \| BFS + DTT | 0.8324 | 0.0358* |
| ATR, s \| BFS - DTT | 0.9475 | 0.6387 |
| EtOH, s \| BFS + DTT | 0.6851 | 0.0006* |
| EtOH s \| BFS - DTT | 0.7197 | 0.0015* |
| DAF, w \| BFS + DTT | 0.9814 | 0.9419 |
| DAF, w \| BFS - DTT | 0.8718 | 0.2737 |
| FD, w \| BFS + DTT | 0.9582 | 0.7957 |
| FD, w \| BFS - DTT | 0.8423 | 0.1715 |
| EtOH, s \| NM | 0.8646 | 0.277 |
| ATR, s \| NM | 0.8555 | 0.2444 |

## Kruskal-Wallis test with Conver-Iman post hoc test

Because non normally distributed data was obtained a nonparametric Kruskal-Wallis test (function kruskal_test from the package rstatix) with Conver-Iman post hoc test (function conover.test from the package conover.test) were chosen for multiple pairwise comparisons between combinations of tested storage conditions and isolation methods for DNA concentrations. The null hypothesis of Kruskal-Wallis test states that the medians of each sample (in our case combination) are the same, meaning that all samples come from the same distribution. For p-values less than 0.05 the the medians of each sample (combination) are not the same and the analysis can be continued with a post hoc test. The Conver-Iman post hoc test tests for median difference in each pairwise comparison of the samples (combinations). P-value of Kruskal-Wallis test for the obtained data was less than 0.05 (χ^2^ = 38.6, p-value = 0.0002), thus we continued with The Conver-Iman test. The obtained p-values of pairwise comparison of the combinations are presented in the table below (Table S2). For p-values less than 0.05 difference in medias in pairwise comparison of the combinations is statistically significant.

**Supplementary Table 2.2.1.** P-values of Conver-Iman post hoc test

|  | ATR, s \| NM | ATR, s \| BFS - DTT | ATR, s \| BFS + DTT | ATR, w \| BFS - DTT | ATR, w \| BFS + DTT | DAF, w \| BFS - DTT | DAF, w \| BFS + DTT | EtOH, s \| NM | EtOH, s \| BFS - DTT | EtOH, s \| BFS + DTT | EtOH, w \| BFS - DTT | EtOH, w \| BFS + DTT | FD, w \| BFS - DTT |
| --- | --- | --- | --- | --- | --- | --- | --- | --- | --- | --- | --- | --- | --- |
| ATR, s \| BFS - DTT | 0.8337 |  |  |  |  |  |  |  |  |  |  |  |  |
| ATR, s \| BFS + DTT | 0.5978 | 0.3304 |  |  |  |  |  |  |  |  |  |  |  |
| ATR, w \| BFS - DTT | 0.0403* | 0.0068* | 0.0509 |  |  |  |  |  |  |  |  |  |  |
| ATR, w \| BFS + DTT | 0.0594 | 0.0121* | 0.0805 | 0.8547 |  |  |  |  |  |  |  |  |  |
| DAF, w \| BFS - DTT | 0.0329* | 0.0050* | 0.0400* | 0.9271 | 0.7836 |  |  |  |  |  |  |  |  |
| DAF, w \| BFS + DTT | 0.1427 | 0.0447* | 0.2182 | 0.5222 | 0.6473 | 0.4647 |  |  |  |  |  |  |  |
| EtOH, s \| NM | 0.4589 | 0.4991 | 0.1598 | 0.0053* | 0.0086* | 0.0041* | 0.0264* |  |  |  |  |  |  |
| EtOH, s \| BFS - DTT | 0.4674 | 0.4939 | 0.0997 | 0.0013* | 0.0025* | 0.0009* | 0.0112* | 0.8736 |  |  |  |  |  |
| EtOH, s \| BFS + DTT | 0.5056 | 0.5462 | 0.1170 | 0.0016* | 0.0030* | 0.0011* | 0.0133* | 0.8257 | 0.9355 |  |  |  |  |
| EtOH, w \| BFS - DTT | 0.0467* | 0.0085* | 0.0607 | 0.9453 | 0.9089 | 0.8727 | 0.5676 | 0.0064* | 0.0017* | 0.0020* |  |  |  |
| EtOH, w \| BFS + DTT | 0.0515 | 0.0098* | 0.0680 | 0.9089 | 0.9453 | 0.8368 | 0.5989 | 0.0072* | 0.0019* | 0.0024* | 0.9635 |  |  |
| FD, w \| BFS - DTT | 0.1998 | 0.0740 | 0.3148 | 0.4024 | 0.5123 | 0.3534 | 0.8428 | 0.0411* | 0.0202* | 0.0238* | 0.4419 | 0.4694 |  |
| FD, w \| BFS + DTT | 0.0029* | 0.0002* | 0.0021* | 0.2977 | 0.2217 | 0.3417 | 0.0949 | 0.0002* | 0.0000* | 0.0000* | 0.2673 | 0.2483 | 0.0626 |

# Qualitative check of the DNA extracts

## Gel electrophoresis of tRNA^leu^-COX2


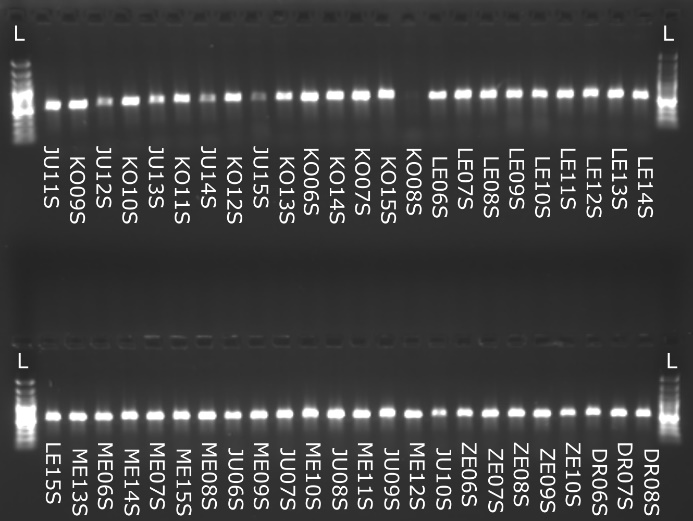

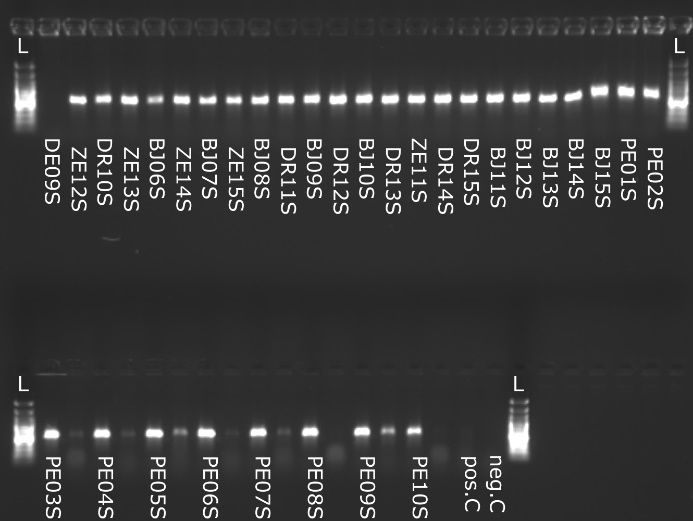


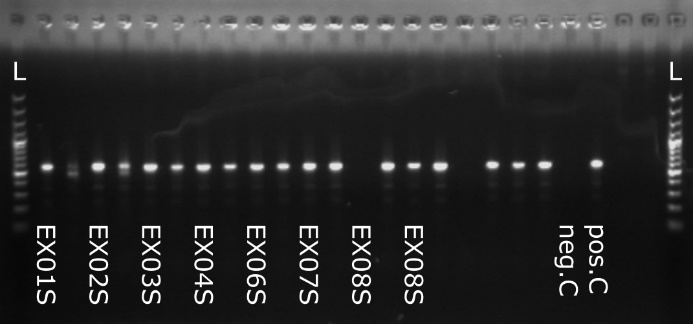


**Supplementary Figures 3.1.1.** Agarose gel electrophoresis of amplified tRNA^leu^-COX2 marker from spermatheca samples. L – 100 bp standard ladder, neg.C – negative control, pos.C - positive control.

## Gel electrophoresis of ANT2


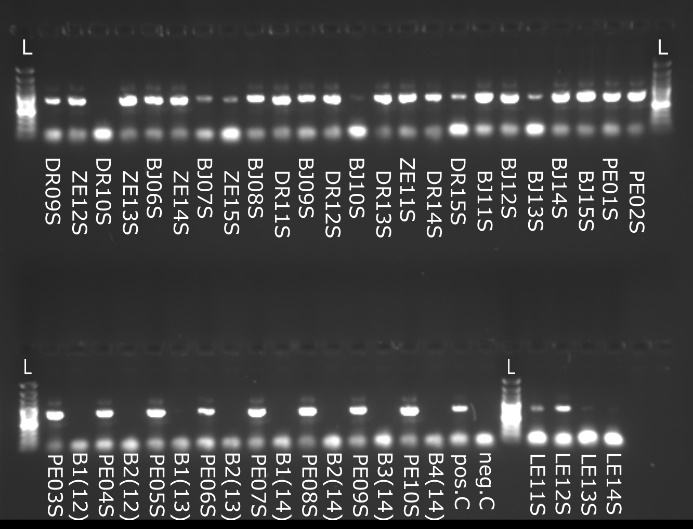

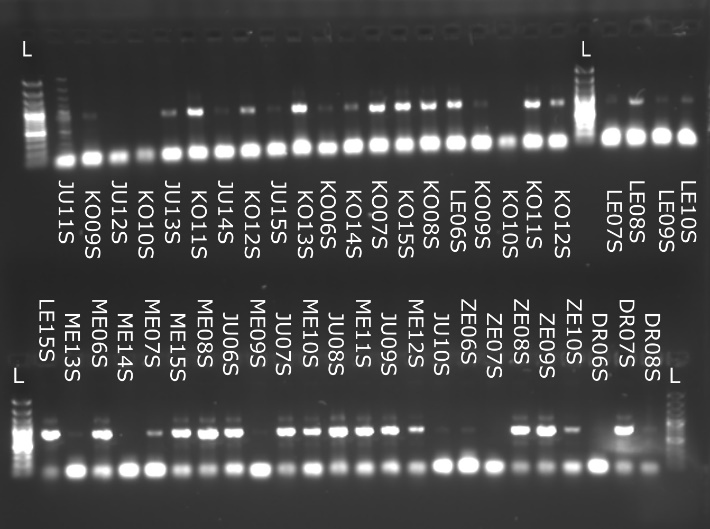


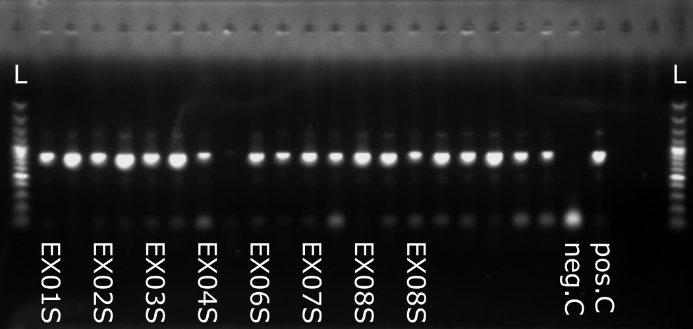

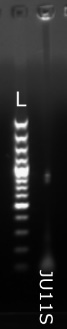


**Supplementary Figures 3.2.1.** Agarose gel electrophoresis of amplified ANT2 marker from spermatheca samples. L – 100 bp standard ladder, neg.C – negative control, pos.C - positive control.

## Success of PCR amplification of mitochondrial tRNAleu-COX2 and nuclear ANT2 markers.

**Supplementary Table 3.3.1.** The numbers represent the quantity of successfully amplified samples in each group. Abbreviations: ATR - Allprotect Tissue Reagent, EtOH - absolute ethanol, DAF - dissected after freezing, DF - freshly dissected, BFS - QIAamp protocol 'Isolation of Total DNA from Body Fluid Stains', + DTT - with dithiothreitol, - DTT - without dithiothreitol, NM - NucleoMag Tissue Kit's protocol.

| **Storage method** | **DNA isolation method** | **tRNA**^leu^**-COX2** | **ANT2** |
| --- | --- | --- | --- |
| Whole, FD | BFS - DTT | 5/5 | 5/5 |
|  | BFS + DTT | 5/5 | 5/5 |
| Whole, DAF | BFS - DTT | 5/5 | 5/5 |
|  | BFS + DTT | 5/5 | 5/5 |
| Whole, EtOH | BFS - DTT | **4/5** | **4/5** |
|  | BFS + DTT | 5/5 | **4/5** |
| Whole, ATR | BFS - DTT | 5/5 | 5/5 |
|  | BFS + DTT | 5/5 | 5/5 |
| Spermatheca, EtOH | BFS - DTT | **9/10** | **8/10** |
|  | BFS + DTT | 10/10 | **8/10** |
|  | NM | 4/4 | 4/4 |
| Spermatheca, ATR | BFS - DTT | 10/10 | **9/10** |
|  | BFS + DTT | 10/10 | 10/10 |
|  | NM | **3/4** | 4/4 |

# Genotypes of successfully amplified microsatellite loci A7 and A113

**Supplementary Table 4.1.** Genotypes of microsatellite **A7** of DNA extracts obtained from different storing conditions of honeybee samples and DNA isolating method from spermathecas. Abbreviations: ATR - Allprotect Tissue Reagent, EtOH - absolute ethanol, DAF - dissected after freezing, DF - freshly dissected, BFS - QIAamp protocol 'Isolation of Total DNA from Body Fluid Stains', + DTT - with dithiothreitol, - DTT - without dithiothreitol, NM - NucleoMag Tissue Kit's protocol.

| **Storage method** | | **DNA isolation method** | **Queens alleles** | | **Alleles in spermatheca** | | | | | | | | |
| --- | --- | --- | --- | --- | --- | --- | --- | --- | --- | --- | --- | --- | --- |
| Spermatheca | ATR | BFS - DTT | 108 | 115 | 102 | 108 | 113 | 115 | 117 | 119 |  |  |  |
|  |  |  | 115 |  | 102 | 108 | 115 | 117 |  |  |  |  |  |
|  |  |  | 108 | 113 | 102 | 108 | 113 | 115 | 117 |  |  |  |  |
|  |  |  | 108 | 115 | 104 | 108 | 113 | 115 |  |  |  |  |  |
|  |  |  | 113 | 115 | 102 | 110 | 113 | 115 | 117 |  |  |  |  |
|  |  | BFS + DTT | 113 | 115 | 102 | 108 | 113 | 115 | 117 |  |  |  |  |
|  |  |  | 113 |  | 102 | 113 | 115 |  |  |  |  |  |  |
|  |  |  | 108 | 113 | 108 | 113 |  |  |  |  |  |  |  |
|  |  |  | 113 | 115 | 102 | 113 | 115 | 117 |  |  |  |  |  |
|  |  |  | 108 | 113 | 108 | 113 | 115 |  |  |  |  |  |  |
|  |  | NM | NA* |  | 102 | 108 | 113 | 115 |  |  |  |  |  |
|  |  |  | 113 | 117 | 102 | 108 | 113 | 115 | 117 |  |  |  |  |
|  |  |  | 113 |  | 102 | 108 | 113 | 115 | 117 |  |  |  |  |
|  |  |  | 108 | 113 | 108 | 113 | 115 |  |  |  |  |  |  |
|  | EtOH | BFS - DTT | 108 | 115 | 108 | 115 | 117 |  |  |  |  |  |  |
|  |  |  | 108 | 113 | 102 | 108 | 113 | 115 | 117 |  |  |  |  |
|  |  |  | 102 | 113 | 102 | 108 | 113 | 115 | 117 | 119 | 131 |  |  |
|  |  |  | 113 | 145 | 102 | 108 | 113 | 115 | 145 |  |  |  |  |
|  |  |  | 113 |  | 102 | 108 | 113 | 115 | 117 |  |  |  |  |
|  |  | BFS + DTT | 111 | 113 | 102 | 108 | 113 | 115 | 117 | 132 | 138 | 148 | 150 |
|  |  |  | 115 |  | 102 | 115 | 117 |  |  |  |  |  |  |
|  |  |  | 108 | 115 | 108 | 113 | 115 |  |  |  |  |  |  |
|  |  |  | 113 | 115 | 102 | 113 | 115 |  |  |  |  |  |  |
|  |  |  | 113 | 115 | 102 | 108 | 113 | 115 | 119 | 127 |  |  |  |
|  |  | NM | 108 | 117 | 102 | 108 | 113 | 115 | 117 | 119 |  |  |  |
|  |  |  | 101 | 115 | 102 | 115 | 127 |  |  |  |  |  |  |
|  |  |  | 115 |  | 102 | 115 |  |  |  |  |  |  |  |
|  |  |  | 108 | 113 | 102 | 108 | 113 | 115 |  |  |  |  |  |

*genotyping was unsuccessful

| Whole queen | ATR | BFS - DTT | 117 | 131 | 113 | 115 | 117 |  |  |  |  |  |  |
| --- | --- | --- | --- | --- | --- | --- | --- | --- | --- | --- | --- | --- | --- |
|  |  |  | 108 | 131 | 108 | 113 | 115 | 117 | 131 |  |  |  |  |
|  |  |  | 115 | 117 | 108 | 110 | 113 | 115 |  |  |  |  |  |
|  |  |  | 108 | 131 | 108 | 113 | 115 | 127 | 131 |  |  |  |  |
|  |  |  | 108 | 115 | 108 | 113 | 115 | 117 |  |  |  |  |  |
|  |  | BFS + DTT | 108 | 117 | 102 | 108 | 113 | 115 | 117 | 119 |  |  |  |
|  |  |  | 110 | 113 | 102 | 113 | 115 | 117 | 119 |  |  |  |  |
|  |  |  | 102 | 113 | 102 | 108 | 113 | 115 |  |  |  |  |  |
|  |  |  | 113 |  | 102 | 108 | 113 | 115 | 119 |  |  |  |  |
|  |  |  | 102 | 113 | 102 | 108 | 110 | 113 | 115 | 119 |  |  |  |
|  | EtOH | BFS - DTT | 108 | 115 | 108 | 115 | 117 |  |  |  |  |  |  |
|  |  |  | 102 | 113 | 102 | 106 | 113 | 115 | 119 |  |  |  |  |
|  |  |  | 108 | 117 | 102 | 113 | 115 | 131 |  |  |  |  |  |
|  |  |  | 108 | 113 | 102 | 108 | 113 | 115 | 117 |  |  |  |  |
|  |  |  | 102 | 113 | 102 | 108 | 113 | 117 | 142 |  |  |  |  |
|  |  | BFS + DTT | 115 |  | 108 | 113 | 115 | 117 | 119 | 141 |  |  |  |
|  |  |  | 110 | 115 | 108 | 110 | 113 | 115 |  |  |  |  |  |
|  |  |  | 108 | 115 | 102 | 108 | 113 | 117 | 127 |  |  |  |  |
|  |  |  | 108 | 115 | 102 | 108 | 113 | 115 | 117 |  |  |  |  |
|  |  |  | 115 |  | 102 | 113 | 115 | 117 | 127 |  |  |  |  |
|  | DAF | BFS - DTT | 104 | 113 | 104 | 113 | 115 | 117 | 141 |  |  |  |  |
|  |  |  | 102 | 113 | 102 | 113 | 115 | 119 | 127 | 131 |  |  |  |
|  |  |  | 113 |  | 113 | 141 |  |  |  |  |  |  |  |
|  |  |  | 113 | 115 | 102 | 108 | 113 | 115 |  |  |  |  |  |
|  |  |  | 108 | 113 | 108 | 113 | 115 | 127 |  |  |  |  |  |
|  |  | BFS + DTT | 113 | 117 | 113 | 115 | 117 | 141 |  |  |  |  |  |
|  |  |  | 108 | 113 | 102 | 108 | 113 | 115 | 127 |  |  |  |  |
|  |  |  | 108 | 113 | 102 | 108 | 113 | 115 | 117 | 141 |  |  |  |
|  |  |  | 110 | 113 | 110 | 113 |  |  |  |  |  |  |  |
|  |  |  | 113 | 115 | 102 | 113 | 115 | 117 | 139 |  |  |  |  |
|  | FD | BFS - DTT | 104 | 115 | 102 | 113 | 115 |  |  |  |  |  |  |
|  |  |  | 108 | 113 | 102 | 108 | 113 | 115 | 117 | 123 | 136 |  |  |
|  |  |  | 113 |  | 108 | 113 | 115 | 117 |  |  |  |  |  |
|  |  |  | 113 |  | 108 | 113 | 115 | 117 |  |  |  |  |  |
|  |  |  | 108 | 115 | 108 | 113 | 115 | 117 |  |  |  |  |  |
|  |  | BFS + DTT | 102 | 113 | 102 | 104 | 113 | 115 | 117 |  |  |  |  |
|  |  |  | 113 |  | 113 | 115 | 117 |  |  |  |  |  |  |
|  |  |  | 113 |  | 113 | 115 | 117 |  |  |  |  |  |  |
|  |  |  | 115 |  | 104 | 113 | 115 | 117 |  |  |  |  |  |
|  |  |  | 115 | 117 | 104 | 108 | 113 | 115 | 117 |  |  |  |  |

**Supplementary Table 4.2.** Genotypes of microsatellite **A24** of DNA extracts obtained from different storing conditions of honeybee samples and DNA isolating method from spermathecas. Abbreviations: ATR - Allprotect Tissue Reagent, EtOH - absolute ethanol, DAF - dissected after freezing, DF - freshly dissected, BFS - QIAamp protocol 'Isolation of Total DNA from Body Fluid Stains', + DTT - with dithiothreitol, - DTT - without dithiothreitol, NM - NucleoMag Tissue Kit's protocol.

| **Storage method** | | **DNA isolation method** | **Queens alleles** | | **Alleles in spermatheca** | | | |
| --- | --- | --- | --- | --- | --- | --- | --- | --- |
| Spermatheca | ATR | BFS - DTT | 102 | 104 | 102 | 104 | 94 |  |
|  |  |  | 104 |  | 104 | 94 |  |  |
|  |  |  | 102 |  | 102 | 104 |  |  |
|  |  |  | 102 | 104 | 102 | 104 | 94 |  |
|  |  |  | 102 | 94 | 102 | 104 | 94 |  |
|  |  | BFS + DTT | 102 | 104 | 102 | 104 | 94 |  |
|  |  |  | 102 | 104 | 102 | 104 | 94 |  |
|  |  |  | 102 | 104 | 102 | 104 | 94 |  |
|  |  |  | 102 | 104 | 102 | 104 | 94 |  |
|  |  |  | 102 | 104 | 102 | 104 | 94 |  |
|  |  | NM | 102 | 104 | 102 | 104 |  |  |
|  |  |  | 102 | 104 | 102 | 104 | 94 |  |
|  |  |  | 102 | 104 | 102 | 104 |  |  |
|  |  |  | 104 |  | 102 | 104 | 94 |  |
|  | EtOH | BFS - DTT | 102 | 104 | 102 | 104 | 94 |  |
|  |  |  | 102 | 104 | 104 |  |  |  |
|  |  |  | 102 |  | 102 | 104 | 94 |  |
|  |  |  | 102 | 104 | 104 | 94 |  |  |
|  |  |  | 102 | 104 | 102 | 104 | 94 |  |
|  |  | BFS + DTT | 104 |  | 104 | 94 |  |  |
|  |  |  | 102 | 104 | 102 | 104 | 94 |  |
|  |  |  | 104 |  | 102 | 104 | 94 |  |
|  |  |  | 104 |  | 104 |  |  |  |
|  |  |  | 102 | 98 | 100 | 102 | 104 |  |
|  |  | NM | 102 | 94 | 102 | 104 | 90 | 94 |
|  |  |  | 102 | 104 | 102 | 104 | 94 |  |
|  |  |  | 102 |  | 102 | 104 | 94 |  |
|  |  |  | 104 |  | 104 | 94 |  |  |

| Whole queen | ATR | BFS - DTT | 104 |  | 102 | 104 | 94 |  |
| --- | --- | --- | --- | --- | --- | --- | --- | --- |
|  |  |  | 102 | 94 | 102 | 104 | 94 |  |
|  |  |  | 102 | 104 | 102 | 104 | 94 |  |
|  |  |  | 102 | 104 | 102 | 104 |  |  |
|  |  |  | 104 |  | 102 | 104 |  |  |
|  |  | BFS + DTT | 102 |  | 102 | 104 |  |  |
|  |  |  | 104 |  | 104 | 94 |  |  |
|  |  |  | 104 |  | 102 | 104 | 94 |  |
|  |  |  | 102 | 104 | 102 | 104 | 94 |  |
|  |  |  | 102 | 104 | 102 | 104 |  |  |
|  | EtOH | BFS - DTT | 104 | 94 | 104 | 94 |  |  |
|  |  |  | 104 |  | 104 | 94 |  |  |
|  |  |  | 102 | 104 | 102 | 104 | 94 |  |
|  |  |  | 102 | 94 | 102 | 104 | 94 |  |
|  |  |  | 102 | 104 | 102 | 104 | 94 |  |
|  |  | BFS + DTT | 102 |  | 102 | 104 | 94 |  |
|  |  |  | 102 | 104 | 102 | 104 |  |  |
|  |  |  | 102 |  | 102 | 104 | 94 |  |
|  |  |  | 102 | 104 | 102 | 104 | 94 |  |
|  |  |  | 102 | 104 | 102 | 104 | 94 |  |
|  | DAF | BFS - DTT | 102 | 104 | 104 | 94 |  |  |
|  |  |  | 104 |  | 104 | 94 |  |  |
|  |  |  | 102 | 104 | 102 | 104 |  |  |
|  |  |  | 102 | 104 | 102 | 104 |  |  |
|  |  |  | 102 | 104 | 102 | 104 | 94 |  |
|  |  | BFS + DTT | 104 |  | 102 | 104 |  |  |
|  |  |  | 102 | 94 | 102 | 104 | 94 |  |
|  |  |  | 102 | 104 | 102 | 104 | 94 |  |
|  |  |  | 102 | 104 | 102 | 104 |  |  |
|  |  |  | 102 | 104 | 102 | 104 |  |  |
|  | FD | BFS - DTT | 102 | 94 | 102 | 94 |  |  |
|  |  |  | 102 | 104 | 102 | 104 | 94 |  |
|  |  |  | 102 |  | 102 | 104 | 94 |  |
|  |  |  | 102 | 104 | 102 | 104 | 94 |  |
|  |  |  | 104 |  | 102 | 104 | 94 |  |
|  |  | BFS + DTT | 104 |  | 102 | 104 | 94 |  |
|  |  |  | 94 |  | 102 | 104 | 94 |  |
|  |  |  | 102 | 94 | 102 | 104 | 94 |  |
|  |  |  | 102 | 104 | 102 | 104 | 94 |  |
|  |  |  | 102 | 94 | 102 | 104 | 94 |  |

# Gel electrophoresis of amplified microsatellite loci A7 and A113


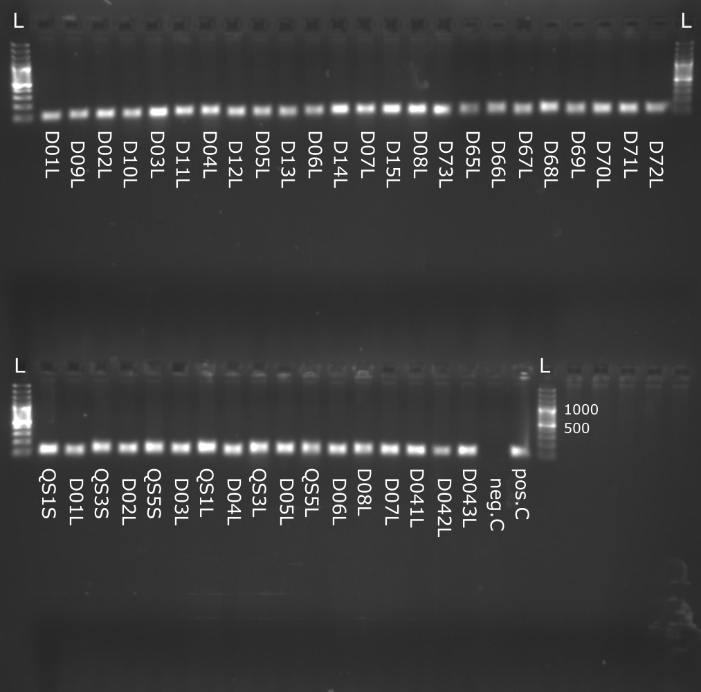

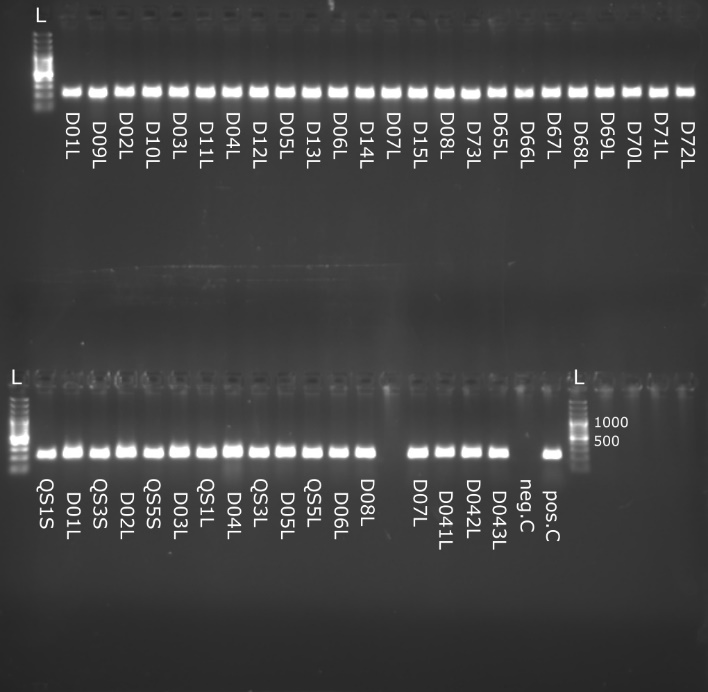


**Supplementary Figures 5.1.** Agarose gel electrophoresis of amplified microsatellite loci A7 (right) and A133 (left) from spermatheca, its corresponding queen and drone samples. L – 100 bp standard ladder, neg.C – negative control, pos.C - positive control.
